# Species Distribution and Prevalence of Putative Virulence Factors in Mesophilic *Aeromonas* spp. Isolated from Fresh Retail Sushi

**DOI:** 10.3389/fmicb.2017.00931

**Published:** 2017-05-24

**Authors:** Sunniva Hoel, Olav Vadstein, Anita N. Jakobsen

**Affiliations:** Department of Biotechnology and Food Science, Norwegian University of Science and TechnologyTrondheim, Norway

**Keywords:** *Aeromonas* spp., virulence factors, *gyrB*, sushi, ready-to-eat food, food safety

## Abstract

*Aeromonas* spp. are ubiquitous bacteria that have received increasing attention as human pathogens because of their widespread occurrence in food, especially seafood and vegetables. The aim of this work was to assess the species identity and phylogenetic relationship of 118 *Aeromonas* strains isolated from fresh retail sushi from three producers, and to characterize the isolates with respect to genetic and phenotypic virulence factors. We also evaluate the potential hazard associated with their presence in ready-to-eat seafood not subjected to heat treatment. Mesophilic *Aeromonas salmonicida* was most prevalent (74%), followed by *A. bestiarum* (9%), *A. dhakensis* (5%), *A. caviae* (5%), *A. media* (4%), *A. hydrophila* (2%), and *A. piscicola* (1%). All isolates were considered potentially pathogenic due to the high prevalence of genes encoding hemolysin (*hlyA*) (99%), aerolysin (*aerA*) (98%), cytotoxic enterotoxin (*act*) (86%), heat-labile cytotonic enterotoxin (*alt*) (99%), and heat-stable cytotonic enterotoxin (*ast*) (31%). The shiga-like toxins 1 and 2 (*stx-1* and *stx-2*) were not detected. Moreover, there was heterogeneity in toxin gene distribution among the isolates, and the combination of *act/alt/hlyA/aerA* was most commonly detected (63%). β-hemolysis was species-dependent and observed in 91% of the isolates. All *A. media* and *A. caviae* strains were non-hemolytic. For isolates belonging to this group, lack of hemolysis was possibly related to the absence of the *act* gene. Swimming motility, linked to adhesion and host invasion, occurred in 65% of the isolates. Partial sequencing of the *gyrB* gene demonstrated its suitability as a genetic marker for *Aeromonas* species identification and for assessment of the phylogenetic relationship between the isolates. The *gyrB* sequence divergence within a given species ranged from 1.3 to 2.9%. *A. bestiarum, A. salmonicida*, and *A. piscicola* were the most closely related species; their sequences differed by 2.7–3.4%. The average *gyrB* sequence similarity between all species was 93%, demonstrating its acceptable taxonomic resolution. The presence of multiple species of potential pathogenic *Aeromonas* in fresh retail sushi raises new food safety issues related to the increased consumption of ready-to-eat food composed of raw ingredients.

## Introduction

*Aeromonas* species are ubiquitous aquatic bacteria that have received increasing attention as opportunistic and primary pathogens in humans. The genus *Aeromonas* can be classified into two main groups. The first is the psychrophilic non-motile strains, primarily *Aeromonas salmonicida*, that infect fish. The second and larger group is motile, mesophilic aeromonads associated with human diseases such as gastrointestinal diseases, wound infections, and septicemia (Janda and Abbott, 2010; Parker and Shaw, 2011). Of the approximately 30 recognized species of *Aeromonas* (Martínez-Murcia et al., 2016), a subset of four species are more frequently implicated in human infections (*Aeromonas hydrophila, A. caviae, A. veronii* biovar *sobria*, and *A. dhakensis*) (Janda and Abbott, 2010; Teunis and Figueras, 2016).

In a previous study, we detected *Aeromonas* spp. in 71% of fresh retail ready-to-eat (RTE) sushi boxes purchased in Norwegian supermarkets (Hoel et al., 2015). These products are offered as complete meals with a selection of different sushi and are distributed cold (≤4°C) with a shelf life of 2–3 days after production. In addition to acidified rice (pH < 4.6), the sushi meals typically consist of various species of both raw fish and vegetables, which are excellent substrates for bacterial growth. The widespread occurrence of *Aeromonas* ssp. in fish and seafood, vegetables, meat, poultry, raw milk, and different water sources has been confirmed by others (Villari et al., 2000; Kingombe et al., 2004; Heaton and Jones, 2008; Xanthopoulos et al., 2010; Ottaviani et al., 2011; Carvalho et al., 2012; Latif-Eugenín et al., 2016).

Most aeromonads grow optimally around 28°C but can grow at a wide range of temperatures (4–42°C) (Palumbo et al., 1985a). Environmental isolates (such as food isolates) seem to be more adapted to low temperatures and can proliferate at refrigeration temperatures (Mateos et al., 1993). Growth at temperatures as low as -0.1°C has been reported for some strains (Daskalov, 2006). Species belonging to the genus *Aeromonas* are generally recognized as sensitive to low pH (min. pH∼6), but food isolates have demonstrated increased tolerability to low pH (Hwang, 2010).

The pathogenesis of *Aeromonas*-mediated infections is multi-factorial, and the role of the virulence determinants in human infections have not been conclusively established (Pablos et al., 2010). An assortment of virulence factors enables these bacteria to colonize, invade, and infect different hosts. The most reviewed virulence factors are the pore-forming hemolytic toxins, hemolysin, and aerolysin, (Howard et al., 1987; Parker and Shaw, 2011), and three different enterotoxins, including cytotoxic (Act), heat-labile cytotonic (Alt), and heat-stable cytotonic (Ast) enterotoxins. All these toxins have been linked to cases of diarrhea (Galindo et al., 2006). Furthermore, the expression of lateral or peritrichous flagella is associated with enhanced adherence and invasiveness in addition to the ability to biofilm formation (Gavín et al., 2003).

The taxonomy of the genus *Aeromonas* is complex and has undergone numerous changes with the implementation of new phylogenetic markers. Conventional phenotypic tests do not necessarily correspond to results achieved by genetic methods, and this is especially evident in environmental isolates of *Aeromonas* species (Ørmen et al., 2005; Puthucheary et al., 2012). Sequencing of the 16S rDNA has proven unsuccessful for identification of *Aeromonas* species due to low taxonomic resolution (Morandi et al., 2005; Nagar et al., 2013). Therefore, sequencing of the housekeeping genes *gyrB* (encoding the B-subunit of DNA gyrase, a type II DNA topoisomerase) (Yanez et al., 2003) and *rpoD* (encoding the σ^70^ factor conferring promoter-specific transcription initiation on RNA polymerase) (Soler et al., 2004) is now preferred for phylogenetic analysis and species identification of *Aeromonas* spp.

The aim of this work was to assess the species identity and phylogenetic relationship of *Aeromonas* strains isolated from fresh retail sushi from three producers and to characterize the isolates with respect to genetic and phenotypic virulence factors. Furthermore, we evaluate the potential hazard associated with their presence in minimally processed RTE seafood such as sushi. To the best of our knowledge, no studies have been published which concurrently assess prevalence, species distribution, and genetic characterization of several *Aeromonas* species in seafood intended for raw consumption. In light of the taxonomical controversies, including recent species reclassifications and the lack of definite virulence markers, studies providing updated characterization of current environmental strains are required in order to provide insight into the impact of these bacteria as foodborne pathogens.

## Materials and Methods

### Bacterial Strains

Mesophilic *Aeromonas* spp. were isolated from retail sushi and verified phenotypically according to Nordic Committee on Food Analysis [NMKL] (2004) method no. 150, as described previously (Hoel et al., 2015) and by PCR using primers targeting the *Aeromonas gyrB* gene (**Table 1**). In brief, *Aeromonas* spp. were isolated from sushi using starch ampicillin agar (SAA) supplemented with 10 mg/L ampicillin (Sigma–Aldrich, Oslo, Norway) and incubated at 37°C. All subsequent cultivation was carried out on tryptone soy agar (TSA) (Oxoid, Oslo, Norway) at 37°C. One hundred and three *Aeromonas* isolates were isolated from sushi products from three different producers: A (*n* = 54), B (*n* = 24), and C (*n* = 25). To increase the resolution in species diversity, 15 additional *Aeromonas* isolates were collected from producer A in a follow-up study. Hence, a total of 118 *Aeromonas* isolates were analyzed. *A. hydrophila* (CCUG 14551^T^), *A. caviae* (CCUG 25939^T^), *A.* veronii biovar *veronii* (CCUG 27821^T^), and *A. veronii* biovar *sobria* (CCUG 30360) were included as reference strains. *Pseudomonas aeruginosa* (CCUG 2080), *Escherichia coli* (CCUG 49263), and *Staphylococcus aureus* (CCUG 41582) were used as negative controls in all PCR reactions to verify the amplification specificity.

**Table 1:** PCR primers, annealing temperatures, and expected amplicon length for *Aeromonas* genus-specific *gyrB* primers and virulence-associated genes.

Target gene	Primer sequence (5′-3′)	Annealing temp (°C)	Amplicon length (nt)	Reference
*gyrB*	GAAGGCCAAGTCGGCCGCCAG	61	198	Tacao et al., 2005
	ATCTTGGCATCGCCCGGGTTTTC			
*gyrB*	TCCGGCGGTCTGCACGGCGT	52	1100	Yanez et al., 2003
	TTGTCGGGGTTGTACTCGTC			
*hlyA*	GGCCGGTGGCCCGAAGATACGGG	61	597	Wong et al., 1998
	GGCGGCGCCGGACGAGACGGG			
*aerA*	GC(A/T)GA(A/G)CCC(A/G)TCTATCC(A/T)G	58	252	Pablos et al., 2009
	TTTCTCCGGTAACAGGATTG			
*act*	GAGAAGGTGACCACCAAGAAGA	58	361	Kingombe et al., 1999
	AACTGACATCGGCCTTGAACTC			
*alt*	TGCTGGGCCTGCGTCTGGCGGT	58	361	Kingombe et al., 2010
	AGGAACTCGTTGACGAAGCAGG			
*ast*	GACTTCAATCGCTTCCTCAACG	58	536	Kingombe et al., 2010
	GCATCGAAGTCACTGGTGAAGC			
*stx-1*	ATAAATTGCCATTCGTTGACTAC	58	180	Paton and Paton, 1998
	AGAACGCCCACTGAGATCATC			
*stx-2*	GGCACTGTCTGAAACTGCTCC	58	255	Paton and Paton, 1998
	TCGCCAGTTATCTGACATTCTG			

### DNA Isolation and PCR Protocols

Total genomic DNA was extracted from overnight cultures grown in tryptone soy broth (TSB) (Oxoid) at 37°C using the protocol for Gram-negative bacteria in the DNeasy Blood and Tissue kit (Qiagen, Oslo, Norway). All PCR reactions were performed with 25 μl reactions containing 1x PCR buffer (1.5 mM MgCl_2_), 200 μM of each nucleotide, 0.2 μM each primer, 2.5 U *Taq* polymerase (Qiagen), and 50–100 ng template DNA. The PCR amplification cycles were as follows: Initial denaturation at 95°C for 15 min, 30 cycles of denaturation at 95°C for 30 s, annealing for 30 s at temperature given in **Table 1**, and extension at 72°C for 60 s, followed by a final extension at 72°C for 7 min. All PCR reactions were performed in duplicate in separate experiments. PCR products were visualized by electrophoresis in a 1.5% agarose gel (SeaKem, Lonza Group Ltd., Basel, Switzerland) in 1x TAE buffer. Two random PCR products of each detected virulence gene (*ast, act, alt, aerA, and hlyA*) were excised from the agarose gel and purified using the GeneJET Gel Extraction Kit (Thermo Scientific, Oslo, Norway) and sequenced (Eurofins Genomics, Ebersberg, Germany) to confirm the amplicon specificity.

### Species Identification and Phylogenetic Analysis

An approximately 1100 nt fragment of the *Aeromonas gyrB* gene was amplified using primers gyrB3F/gyrB14R (**Table 1**). PCR products were purified using the GeneJet PCR Purification Kit (Thermo Scientific). Eurofins Genomics performed the DNA sequencing, and the resulting sequences were compared with available GenBank database sequences using the BLAST program^1^.

Multiple sequence alignments were performed using ClustalW, integrated in the MEGA7 software (Kumar et al., 2016). The *gyrB* sequences from all isolates and their corresponding type or reference sequences were included in the alignment, but identical sequences were not included in the phylogenetic analysis. The reference or type sequences for the *gyrB* gene for species not available as cultures at our laboratory were retrieved from NCBI: *A. salmonicida* strain CECT 894^T^ (GeneBank acc. No. AY101773), *A. bestiarum* strain CDC 9533-76^T^ (AJ868362), *A. media* strain CECT 4232^T^ (AY101782), *A. dhakensis* strain MDC 2406^T^ (HQ442711) and strain MDC 401 (EU268453), *A. piscicola* strain S1.2^T^ (AY011790) and *E. coli* strain KCTC 2441 (EU014649).

A phylogenetic tree was constructed using the neighbor-joining method (Saitou and Nei, 1987) in the MEGA 7 software, and evolutionary distances were calculated using the Kimura two-parameter method (Kimura, 1980) with 1000 bootstrap replicates to assess tree topology robustness. To test the tree stability, we also created a phylogenetic tree using the maximum-likelihood method. The phylogenetic analysis involved 38 nucleotide sequences with a continuous stretch of 944 nt. Thirty-four partial *gyrB* nucleotide sequences were submitted to the GenBank database^2^ with accession numbers KY652231–KY652264.

### Phenotypic Characterization

To test for β-hemolytic activity, one colony of each isolate was streaked onto bovine blood agar and incubated at 37°C. Hemolysis was recorded after 24 and 48 h (± 2 h) and done twice for each isolate.

Swimming (defined as flagella-directed movement in an aqueous environment) and swarming (defined as multiple, lateral, flagella-directed rapid movements on a solid surface) (Jahid et al., 2015) motility were examined using modifications of methods described by Grim et al. (2013) and Jahid et al. (2013). For swimming motility, a single colony from an exponentially growing culture was spotted into the center of a plate containing Nutrient Agar (3 g/L beef extract [Difco, Becton, Dickinson & Company, Franklin Lakes, NJ, USA], 5 g/L bacterial peptone [Oxoid], and 0.3% agar [Oxoid]). The swimming plates were incubated face up at 25°C for 24 ± 2 h. The test was performed twice for each isolate. For swarming motility, two different methods were applied. First, material from the periphery of the swimming zone was selected and streaked onto the surface of an agar plate containing LB (10 g/L bacterial peptone, 10 g/L NaCl, 5 g/L yeast extract [Oxoid], and 0.5% agar [Oxoid]). Second, a single colony from an overnight culture on TSA was streaked onto the surface of an LB plate containing 0.5% agar. In both cases, plates were incubated at 37°C for 24 and 48 h (± 2 h). Using both methods, an incubation temperature of 25°C was also tested once for all isolates. After incubation, the motility diameter was measured by examining bacterial migration through the agar from the center toward the periphery of the plate.

### Statistical Analysis

A chi-square test was applied to test for differences in species distribution among the three producers and to test for variability in species distribution over time for producer A (α = 0.05) using the online resources for the textbook by Barnard et al. (2011)^3^.

## Results

### *Aeromonas* Species Identification

All presumptive *Aeromonas* isolates (*n* = 118) were verified as *Aeromonas* spp. using genus-specific primers targeting *gyrB*. Nucleotide sequences of *gyrB* amplicons were determined for 118 isolates and 4 *Aeromonas* reference strains. The resulting sequences were 701–1048 nt with 93% > 1030 nt. The prevalence and distribution of species from the initial sampling from producers A, B, and C, and the follow-up sampling of producer A are shown in **Table 2**. There were no differences in the relative occurrence of species between the three producers (*p* = 0.686), and mesophilic (non-typical) *A. salmonicida* was the most commonly isolated species from all producers. However, in the follow-up study of producer A, two more species were detected, and *A. dhakensis*, synonymous with *A. hydrophila* ssp. *dhakensis*/*A. aquariorum*, was the most prevalent. Thus, there was a significant difference in the occurrence of species over time for producer A (*p* < 0.001).

**Table 2:** Number (percentage) of *Aeromonas* species sampled from three sushi producers (A, B, and C), and a follow-up sampling from producer A.

	*n*	*A. salmonicida*	*A. bestiarum*	*A. caviae*	*A. media*	*A. piscicola*	*A. dhakensis*	*A. hydrophila*
Producer A	54	43 (79.5)	8 (14.5)	1 (2)	1 (2)	1 (2)	0	0
Producer B	24	21 (87.5)	3 (12.5)	0	0	0	0	0
Producer C	25	23 (92)	0	0	2 (8)	0	0	0
Follow-up A	15	0	0	5 (33)	2 (13.5)	0	6 (40)	2 (13.5)
Total prevalence	118	87 (74)	11 (9)	6 (5)	5 (4)	1 (1)	6 (5)	2 (2)

### Prevalence of Virulence-Associated Genes

The presence of seven virulence-associated genes (**Table 3**) was analyzed by PCR. At least one of these genes was present in all *Aeromonas* isolates. The reference strains *A. hydrophila* (CCUG 14551) and *A. veronii* biovar *veronii* (CCUG 27821) harbored all genes, except *stx-1* and *stx-2*. *A. veronii* biovar *sobria* (CCUG 30360) harbored *alt, ast, aerA*, and *hlyA*, whereas only the *alt* gene was detected in the *A. caviae* reference strain (CCUG 25939). The non-*Aeromonas* strains (*E. coli, P. aeruginosa*, and *S. aureus*) gave negative results for all genes, except *S. aureus*, which exhibited a very faint band for *hlyA*.

**Table 3:** Virulence associated genes, β-hemolysis on blood agar, and swimming motility in soft agar for *Aeromonas* spp. isolated from retail sushi.

	Number (percentage) of isolates harboring virulence associated genes and phenotypic traits									
Species	*n*	*act*	*alt*	*ast*	*aerA*	*hlyA*	*stx-1*	*stx-2*	β-hemolysis	Swimming motility
*A. salmonicida*	87	87 (100)	87 (100)	19 (22)	87 (100)	87 (100)	0	0	87 (100)	62 (71)
*A. bestiarum*	11	11 (100)	11 (100)	3 (27)	11 (100)	11 (100)	0	0	11 (100)	7 (54)
*A. dhakensis*	6	0	6 (100)	6 (100)	6 (100)^a^	6 (100)	0	0	6 (100)	3 (50)
*A. caviae*	6	0	5 (83)	3 (50)	4 (67)^a^	5 (83)	0	0	0	2 (33)
*A. media*	5	0	5 (100)	2 (40)	4 (80)^a^	5 (100)	0	0	0	1 (20)
*A. hydrophila*	2	2 (100)	2 (100)	2 (100)	2 (100)	2 (100)	0	0	2 (100)	2 (100)
*A. piscicola*	1	1 (100)	1 (100)	1 (100)	1 (100)	1 (100)	0	0	1 (100)	0
Total	118	101 (86)	117 (99)	36 (31)	115 (98)	117 (99)	0	0	107 (91)	77 (65)

*^a^ Faint band, but considered as a positive result.*

**FIGURE 1:**
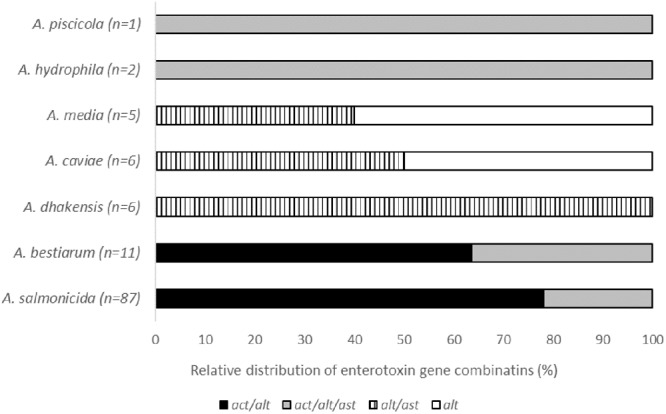
**Relative distribution of the combinations of the enterotoxin genes *act, alt*, and *ast* in the different groups of *Aeromonas* spp**.

Four different combinations of the *act/alt/ast* genes encoding *Aeromonas* enterotoxins were observed. The most frequent enterotoxin gene combination was *act/alt*, present in 75 isolates (64%), followed by *act/alt/ast* in 26 isolates (22%), and *alt/ast* in 11 isolates (9%). A single gene (*alt*) was detected in six isolates (5%), and they were all *A. media* and *A. caviae* species. A species-dependent heterogeneity in the enterotoxin gene distribution was observed (**Figure 1**). The most frequent enterotoxin gene combination *act/alt* was only observed in *A. salmonicida* and *A. bestiarum* isolates. The combination *act/alt/ast* was found in *A. salmonicida, A. bestiarum, A. piscicola*, and A. *hydrophila* isolates, whereas the *alt/ast* and *alt* dominated in the other species.

Genes encoding the hemolytic and pore-forming toxins hemolysin and aerolysin were detected in 99 and 98% of all isolates, respectively (**Table 3**). However, the results for *aerA* were ambiguous for the isolates identified as *A. caviae, A. media*, and *A. dhakensis*. PCR yielded faint bands, regardless of DNA template concentration and attempts to optimize PCR running conditions. However, faint bands were considered positive results. All isolates gave negative results for the *stx-1* and *stx-2* genes, encoding shiga-like toxins 1 and 2, respectively. Sequencing of two PCR products from each gene confirmed their specificity, and the non-template controls were negative for all genes for all samples.

### Hemolytic Activity and Swimming Motility

β-hemolysis was observed in 91% of the isolates, and the outcome of this test was clearly related to species identity (**Table 3**). The non-hemolytic isolates belonged to the species *A. media* and *A. caviae*. In fact, all *A. media* and *A. caviae* in this study were non-hemolytic, including the *A. caviae* reference strain.

Isolates with a circular migration zone surrounding the point of inoculation on the soft agar plates were classified as motile. In cases in which growth was limited to the inoculation point, or the diameter of the growth was ≤10 mm, the isolates were classified as non-motile under the tested conditions, and 41 isolates (35%) were assigned to this group (**Table 3**). Some intra-species variability was observed. All species had both motile and non-motile isolates except *A. piscicola*, which was represented by only one non-motile isolate. Interestingly, most of the non-motile *A. salmonicida* were isolated from producer C’s sushi. We were not able to induce swarming motility in any isolates or reference strains under the tested conditions.

### Phylogenetic Analysis of *Aeromonas gyrB* Sequences

The *gyrB* sequences from all *Aeromonas* isolates and the reference strain sequences were included in the multiple sequence alignment, and a distance matrix was created for all pairs of sequences for identification and removal of duplicate sequences. The sequence similarity between all strains in the final dataset ranged from 90.1 to 99.9 % (1–93 nucleotide differences).

Percentage nucleotide substitutions were calculated for a continuous segment of 944 nt (Supplementary Table A). At the intra-species level (between isolates of a given species), the average nucleotide substitution ranged from 1.3 to 2.9% and were <2% for the majority of species (1.3% for *A. bestiarum* and *A. piscicola*, 1.4% for *A. hydrophila*, 1.6% for *A. salmonicida*, and 1.9% for *A. caviae*), with the exception of *A. dhakensis* and *A. media* (2.3 and 2.9%, respectively).

The inter-species sequence divergence was higher and >3.4% for all pairs of species (**Table 4**). The only exception was a tighter group of three species consisting of *A. salmonicida, A. bestiarum*, and *A. piscicola* with nucleotide substitutions ranging from 2.7 to 3.4% (2.7% for *A. piscicola* and *A. bestiarum*, 3.0% for *A. piscicola* and *A. salmonicida*, and 3.4% for *A. bestiarum* and *A. salmonicida*).

**Table 4:** Comparison of average *gyrB* sequence evolutionary divergence (percentage nucleotide substitutions) between the seven *Aeromonas* spp. isolated from retail sushi.

Species	1	2	3	4	5	6
(1) *A. piscicola*						
(2) *A. dhakensis*	8.8					
(3) *A. bestiarum*	2.7	8.9				
(4) *A. caviae*	9.1	5.9	9.4			
(5) *A. hydrophila*	8.3	4.9	8.3	5.2		
(6) *A. media*	7.1	7.3	6.8	7.7	6.9	
(7) *A. salmonicida*	3.0	9.2	3.4	9.3	8.2	7.3

**FIGURE 2:**
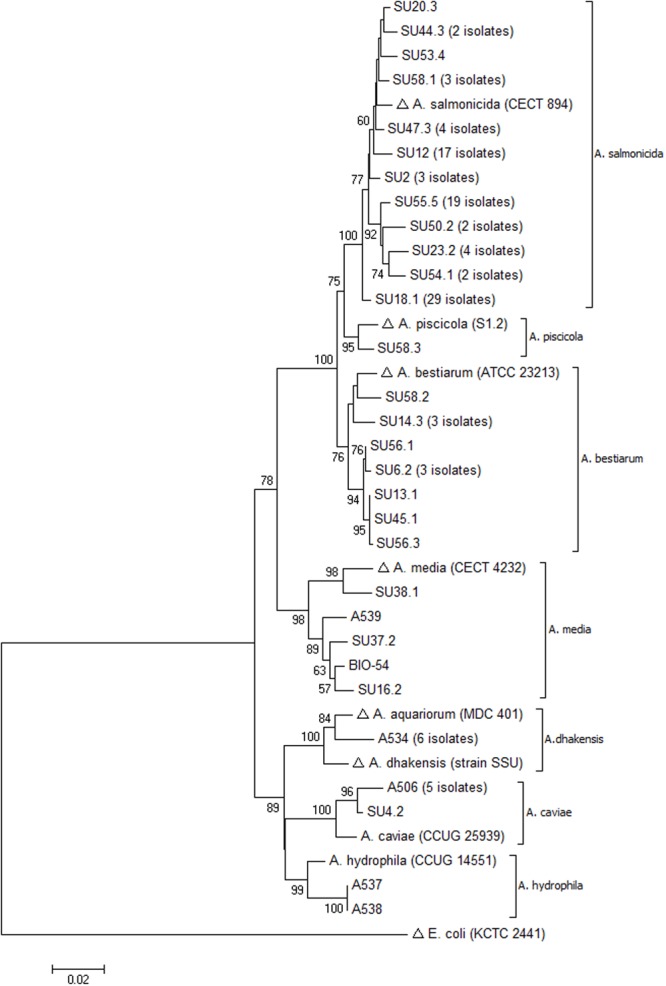
**Neighbor-joining tree based on *gyrB* sequences showing inter-and intra-species evolutionary relationships of representative *Aeromonas* spp. isolated from sushi (with number of identical sequences indicated)**. Sequences retrieved from the GenBank database are marked with (Δ). The percentage of 1000 bootstrap replicates is shown next to the branches (only values > 50% are shown). The scale bar indicates evolutionary distance of 0.02 nucleotide substitutions per site. The analysis involved 38 sequences and a continuous stretch of 944 nt.

A neighbor-joining tree was constructed from the nucleotide alignment, and the constructed tree showed distinct clustering of all species with high bootstrap values (>75%) (**Figure 2**). All isolates clustered with their respective reference or type strain sequences. Moreover, the constructed phylogenetic tree demonstrated branching into two main clusters. One subsection included the species *A. dhakensis, A. hydrophila*, and *A. caviae* whereas the other main cluster included *A. salmonicida, A. piscicola, A. bestiarum*, and *A. media*. Overall, a nearly identical clustering was obtained by phylogenetic analysis using the maximum-likelihood method, confirming the robustness of the neighbor-joining tree topology (data not shown).

## Discussion

In this study, *Aeromonas* strains isolated from retail sushi products from three producers were identified at species level and characterized genetically and phenotypically with respect to virulence traits, hemolysis and motility. We identified seven different *Aeromonas* species, equally distributed between the three producers, with the exception of *A. hydrophila* and *A. dhakensis*, which were isolated from one producer a year after the initial sampling. The variability of species distribution over time for producer A demonstrates the microbiological complexity of these products based on an assortment of raw ingredients. The most frequently isolated species, *A. salmonicida*, is by definition known as a psychrophilic, non-motile species, associated with fish disease (Austin et al., 1998). However, the *A. salmonicida* isolates in the present study fall into a group of so-called non-typical *A. salmonicida* characterized as mesophilic, sometimes motile, bacteria with no or reduced pigment production (Dallaire-Dufresne et al., 2014). Infections attributed to non-typical *A. salmonicida* are an increasing problem in aquaculture, mainly affecting the cleaner fish (wrasse) (Hjeltnes et al., 2015; Biering et al., 2016). A high prevalence of mesophilic *A. salmonicida* was also reported in studies of Italian food samples (Ottaviani et al., 2011) and in Mexican frozen fish samples (Castro-Escarpulli et al., 2003).

Phylogenetic analysis of *Aeromonas* spp. based on 16S rDNA sequencing has revealed that the genus is composed of a very tight group of species, some of them differing by only a few nucleotides (Martinez-Murcia et al., 1992; Kupfer et al., 2006). As an example, the inter-species similarity for the 16S rDNA sequences of *A. salmonicida, A. bestiarum*, and *A. piscicola* were reported as high as 99.8–100%, and the species were thus impossible to separate by 16S analysis (Figueras et al., 2011). Moreover, the overall mean 16S rDNA sequence similarity for *Aeromonas* spp. was 97.3%, highlighting the poor discriminatory power of the 16S rRNA gene (Nagar et al., 2013). The discriminatory power of some housekeeping genes was reported to be considerably higher than 16S rDNA, as demonstrated by an 89 and 92% mean sequence similarity for *rpoD* and *gyrB*, respectively (Soler et al., 2004; Nagar et al., 2013). The mean *gyrB* sequence similarity in the present study was 93%, and thus is in accordance with previous studies. The range of nucleotide substitutions within the tighter group of species *A. salmonicida, A. bestiarum*, and *A. piscicola* (2.7–3.4%) was on the border between the values of intra- and inter-species sequence divergence observed here, as reported previously (Yanez et al., 2003). Still, the sequence divergence between the three species were higher than the overall intra-species value (1.8%), demonstrating the power of *gyrB* to separate these phylogenetically close species.

The phylogenetic analysis based on partial *gyrB* sequences resulted in neighbor-joining and maximum-likelihood trees with robust and well-separated clusters of species. Of particular interest, all *A. salmonicida* and *A. bestiarum* isolates were separated into two robust clusters with a 100% bootstrap value (**Figure 2**). Isolate SU58.3 formed a separate sub-branch between the *A. salmonicida* and *A. bestiarum* clusters. Comparison with its closest relatives in the GenBank database produced significant alignment with one sequence of *A. pisciciola* (type strain sequence) and several sequences of *A. bestiarum.* However, isolate SU58.3 clustered with the type strain sequence of *A. piscicola* and their sequence similarity was 98.9%.

The taxonomical controversy of the genus *Aeromonas* was further demonstrated by the recent reclassification of *A. hydrophila* subsp. *dhakensis* (Huys et al., 2002) and *A. aquariorum* (Martinez-Murcia et al., 2008) as *A. dhakensis* (Beaz-Hidalgo et al., 2013). A nucleotide database search with the *gyrB* sequences from the six *A. dhakensis* isolates included in our study resulted mainly in alignments with sequences annotated as *A. hydrophila*. In fact, it is estimated that 30% of the genomes deposited in the GenBank database under the name *A. hydrophila* do not belong to this species (Teunis and Figueras, 2016). However, the phylogenetic analysis of the obtained *gyrB* sequences demonstrated that the six isolates clustered strongly with known sequences of *A. aquariorum*/*A. dhakensis* (**Figure 2**), and they were clearly separated from the *A. hydrophila* cluster. For decades, *A. dhakensis* has been mistaken for *A. hydrophila*, which might have contributed to an overestimation of the clinical significance of *A. hydrophila*. Increasing evidence indicates that *A. dhakensis* is widely distributed in the environment and must be recognized as a potent human pathogen (Chen et al., 2016).

The *Aeromonas* isolates in the present study were considered potentially pathogenic due to the high frequency of virulence-related genes. *Aeromonas* pathogenicity is associated with numerous virulence factors, but there is no definite link between the presence of specific toxin genes and clinical presentation. The lack of classification as a true pathogen is also related to the low number of registered outbreaks of foodborne disease linked to *Aeromonas* spp. Nevertheless, several studies have established an epidemiological link between the sources of infection and clinical isolates of *Aeromonas* spp. (Khajanchi et al., 2010; Pablos et al., 2011; Teunis and Figueras, 2016). As for other opportunistic pathogens, infections by potentially pathogenic *Aeromonas* may not always lead to disease due to host responses and the infectious bacterial dose. However, studies of clinical isolates from patients with various infections have brought more attention to some virulence factors, including the enterotoxins, hemolysins, and their secretion systems, and the clinical *Aeromonas* isolates have been reported to harbor a variety of toxin gene profiles (Rasmussen-Ivey et al., 2016). Furthermore, several studies have effaced the differences between clinical and environmental strains with respect to pathogenicity. The prevalence of toxin genes in isolates from food, environmental, and clinical sources was compared by Ottaviani et al. (2011), and no differences between these groups were detected. In agreement with previous studies of clinical and environmental isolates (Sen and Rodgers, 2004; Khajanchi et al., 2010), we detected multiple virulence-related genes in all sushi isolates. Moreover, we found heterogeneity in the distribution of toxin genes among the isolates, also within species. In our study, the combination of *act*/*alt*/*hlyA*/*aerA* was the most common, observed in 63% of the isolates. Interestingly, we observed variability in the combination of enterotoxin genes between species, but some of the species were represented by only one or a few isolates. In accordance with previous studies, we demonstrated lack of or reduced prevalence of the classical virulence genes in *A. caviae* (Kingombe et al., 1999; Carvalho et al., 2012). A significant proportion of clinical *A. caviae* strains that lacked all the toxin genes or harbored only the *aerA* gene alone were reported (Ottaviani et al., 2011). However, *A. caviae* is considered as one of the most potent human pathogen belonging to the genus *Aeromonas* and is frequently associated with infections (Pablos et al., 2010; Puthucheary et al., 2012). This suggests that for this species, there is a possible involvement of other virulence factors, and it highlights the relevant role of the aerolysin toxin in pathogenesis (Aguilera-Arreola et al., 2007; Ottaviani et al., 2011).

The *act* gene encoding a cytotoxic enterotoxin has been shown to be highly prevalent in strains from drinking water (70%) and seafood (75%) (Sen and Rodgers, 2004; Yano et al., 2015). For this gene, we observed a species-dependent prevalence consisting of a 100% prevalence in *A. salmonicida, A. bestiarum, A. hydrophila*, and *A. piscicola* (represented by only one isolate). In contrast, the *act* gene was not detected in any *A. dhakensis, A. caviae*, or *A. media* isolates. Act and aerolysin are pore-forming toxins, and the hemolytic, cytotoxic, and enterotoxic activities of these proteins have been previously demonstrated (Xu et al., 1998; Buckley and Howard, 1999; Sha et al., 2002). The vast majority of *Aeromonas* isolated from sushi in our study harbored both the *aerA* and *hlyA* genes, but not all of these isolates displayed β-hemolysis on blood agar. Shared characteristics of the non-hemolytic isolates were the absence of *act*, the faint *aerA* band observed on the agarose gel, and the presence of *hlyA.* For these isolates, a link between the absence of the *act* gene and the observed lack of hemolysis is suggested. In contrast, all *A. dhakensis* (*act* negative) isolates in the present study displayed strong hemolysis, suggesting that hemolysis cannot be attributed to a single factor. Wong et al. (1998) proposed a two-toxin model for *aerA* and *hlyA*, in which both genes must be knocked out to reduce virulence. The vast majority of *Aeromonas* isolated from sushi in our study harbored both the *aerA* and *hlyA* genes, but not all of these isolates displayed β-hemolysis on blood agar. Thus, the lack of observed hemolysis might be a result of altered gene expression and/or posttranslational processes not analyzed here.

Regardless of geographical origin, the *alt* and *ast* genes seem to be less prevalent, and their impact on pathogenesis has not been completely recognized. In the present study, the proportion of isolates harboring these genes were higher than reported previously (Di Pinto et al., 2012; Senderovich et al., 2012; Yano et al., 2015). It has been hypothesized that the species *A. caviae* and *A. veronii* lack the *ast* gene (Puthucheary et al., 2012). However, three out of six *A. caviae* isolates from the present study harbored this gene. Moreover, we detected *ast* in the *A. veronii* biovar *veronii* reference strain. Keeping in mind the heterogeneous distribution of various virulence genes in strains from various sources and geographical origin and the possibility of horizontal gene transfer, it is unlikely that the lack of specific genes can be attributed to certain species.

Human *Aeromonas-*mediated infections have occasionally been associated with hemolytic uremic syndrome (HUS), which can lead to acute renal failure and death. HUS is mainly associated with shiga-like toxins (Stx1 and/or Stx2) produced from enterohemorrhagic *E. coli* strains (EHEC) (Figueras et al., 2007). These toxins are encoded by *stx* genes in the genome of a lysogenic bacteriophage (Stx phage) of EHEC and represent a horizontal transfer mechanism (Palma-Martínez et al., 2016). The *stx-1* and *stx-2* genes have been detected in clinical and environmental isolates of *A. hydrophila, A. caviae*, and *A. veronii* biovar *sobria* (Snowden et al., 2006; Alperi and Figueras, 2010; Palma-Martínez et al., 2016). None of our isolates was positive for the *stx-1/-2* genes, and to our knowledge, these genes have not been detected in foodborne isolates.

Bacterial motility, enabled by a lateral or polar flagella, have a number of biological functions in pathogens such as chemotaxis, adhesion, and invasion as seen in *E. coli, P. aeruginosa*, and *Clostridium difficile* (Haiko and Westerlund-Wikström, 2013; Qin et al., 2016). A recent study demonstrated that flagellar motility is necessary for *A. hydrophila* to adhere to the host mucus (Qin et al., 2016). We were able to demonstrate motility on soft agar plates in a significant proportion of *A. salmonicida* (71%) and *A. hydrophila* (100%) isolates, and less in the other species. However, it must be noted that perturbations in the motility assays such as inoculum state, incubation temperature, and brand of agar can give false negative results (Grim et al., 2013).

Most modern approaches for controlling levels of food contamination by microorganisms are effective against *Aeromonas* spp. (Daskalov, 2006). However, the increased consumption of raw or mildly processed RTE food poses some new safety concerns because the food is intended for consumption without further preparation such as washing or heat treatment. We have previously reported relatively high numbers of *Aeromonas* spp. (>log 4 CFU/g) in retail sushi before its stated expiration date. The risk of acute gastrointestinal disease associated with concentrations of *Aeromonas* spp. found in different water and food matrices was recently simulated by Teunis and Figueras (2016). Of particular concern, the concentration of *Aeromonas* spp. in the salad identified as the source of a substantial Chinese outbreak was estimated to be in the range of log 2.3–3.0 CFU/g. Several studies have reported the proliferation of *Aeromonas* spp. at low temperatures (Palumbo et al., 1985b; Palumbo and Buchanan, 1988; Mano et al., 2000; Papageorgiou et al., 2006). However, most studies were done in laboratory cultures or in products not comparable to sushi. Little information is available on the growth rates of *Aeromonas* spp. in sushi during refrigerated storage. Moreover, fluctuations in temperature are likely to occur during production, distribution, and display in stores. We have seen that the growth rates of other bacterial groups increased in sushi during storage at 8°C compared with storage at 4°C (submitted manuscript). Furthermore, analysis of sushi ingredients revealed that *Aeromonas* spp. can be introduced into the product through both raw vegetables and fish (Hoel et al., 2015). In the present study, we isolated *Aeromonas* spp. from a homogenized product containing two species of fish and vegetables (spring onion). Considering the number of species detected, and the fact that all isolates were mesophilic, these bacteria probably originated from more than one ingredient.

## Conclusion

Our results demonstrate that potentially pathogenic *Aeromonas* species are widespread in retail sushi products. The isolates were assigned to seven different species according to *gyrB* sequencing. All isolates harbored several virulence genes, and a significant proportion were hemolytic and motile, thus showing virulence properties comparable to those in clinical strains (Wu et al., 2007; Puthucheary et al., 2012; Senderovich et al., 2012). The results highlight the potential microbiological hazard linked to the presence of *Aeromonas* spp. in sushi and in other perishable RTE food not subjected to heat treatment. Because of the multi-factorial virulence, a rapid discrimination between pathogenic and non-pathogenic strains remains a challenge for the genus *Aeromonas*.

## Author Contributions

SH performed the experiments, phylogenetic analyses, and wrote the manuscript. OV did the statistical analysis, contributed to experimental planning and critically commented and revised the manuscript. AJ contributed to experimental planning and critically commented and revised the manuscript.

## Conflict of Interest Statement

The authors declare that the research was conducted in the absence of any commercial or financial relationships that could be construed as a potential conflict of interest.

## Footnotes

1 http://blast.ncbi.nlm.nih.gov/

2 https://www.ncbi.nlm.nih.gov/genbank/

3 http://www.pearsoned.co.uk/HigherEducation/Resources/BarnardAskingQuestionsinBiology4e/
