# Supplementary material for: Species Distribution and Prevalence of Putative Virulence Factors in Mesophilic Aeromonas spp. Isolated from Fresh Retail Sushi
Source: Front Microbiol. 2017 May 24;8:931. doi: 10.3389/fmicb.2017.00931 (PMC5442234; doi:10.3389/fmicb.2017.00931)
Supplement: Supplementary file 1 [file Table_1.DOCX]

**Supplementary table A** Comparison of nucleotide substitution (%) for *gyrB* sequences (944 nt) of *Aeromonas* isolates and type strains.

| **Strains** | **1** | **2** | **3** | **4** | **5** | **6** | **7** | **8** | **9** | **10** | **11** | **12** | **13** | **14** | **15** | **16** | **17** | **18** | **19** | **20** | **21** | **22** | **23** | **24** | **25** | **26** | **27** | **28** | **29** | **30** | **31** | **32** | **33** | **34** | **35** | **36** | **37** |
| --- | --- | --- | --- | --- | --- | --- | --- | --- | --- | --- | --- | --- | --- | --- | --- | --- | --- | --- | --- | --- | --- | --- | --- | --- | --- | --- | --- | --- | --- | --- | --- | --- | --- | --- | --- | --- | --- |
| 1. *A. piscicola* (S1.2) |  |  |  |  |  |  |  |  |  |  |  |  |  |  |  |  |  |  |  |  |  |  |  |  |  |  |  |  |  |  |  |  |  |  |  |  |  |
| 2. *A. aquariorum* (MDC 401) | 8.4 |  |  |  |  |  |  |  |  |  |  |  |  |  |  |  |  |  |  |  |  |  |  |  |  |  |  |  |  |  |  |  |  |  |  |  |  |
| 3. *A. bestiarum* (ATCC 23213) | 3.0 | 8.7 |  |  |  |  |  |  |  |  |  |  |  |  |  |  |  |  |  |  |  |  |  |  |  |  |  |  |  |  |  |  |  |  |  |  |  |
| 4. *A. caviae* (CCUG 25939) | 8.7 | 5.6 | 8.7 |  |  |  |  |  |  |  |  |  |  |  |  |  |  |  |  |  |  |  |  |  |  |  |  |  |  |  |  |  |  |  |  |  |  |
| 5. Isolate A534 | 9.1 | 2.2 | 9.5 | 6.9 |  |  |  |  |  |  |  |  |  |  |  |  |  |  |  |  |  |  |  |  |  |  |  |  |  |  |  |  |  |  |  |  |  |
| 6. *A. dhakensis* (strain SSU) | 8.3 | 2.3 | 8.0 | 6.0 | 2.6 |  |  |  |  |  |  |  |  |  |  |  |  |  |  |  |  |  |  |  |  |  |  |  |  |  |  |  |  |  |  |  |  |
| 7. *A. hydrophila* (CCUG 14551) | 7.4 | 4.3 | 7.1 | 4.6 | 5.1 | 3.9 |  |  |  |  |  |  |  |  |  |  |  |  |  |  |  |  |  |  |  |  |  |  |  |  |  |  |  |  |  |  |  |
| 8. *A. media* (CECT 4232) | 8.2 | 6.8 | 7.5 | 7.5 | 8.6 | 7.5 | 6.7 |  |  |  |  |  |  |  |  |  |  |  |  |  |  |  |  |  |  |  |  |  |  |  |  |  |  |  |  |  |  |
| 9. *A. salmonicida* (CECT 894) | 3.4 | 9.3 | 3.8 | 8.4 | 9.5 | 8.9 | 7.5 | 8.1 |  |  |  |  |  |  |  |  |  |  |  |  |  |  |  |  |  |  |  |  |  |  |  |  |  |  |  |  |  |
| 10. Isolate A506 | 9.8 | 5.5 | 9.9 | 2.4 | 6.2 | 5.9 | 5.0 | 8.2 | 9.8 |  |  |  |  |  |  |  |  |  |  |  |  |  |  |  |  |  |  |  |  |  |  |  |  |  |  |  |  |
| 11. Isolate A537 | 8.5 | 4.5 | 8.1 | 5.2 | 6.0 | 4.9 | 2.2 | 7.2 | 8.7 | 5.7 |  |  |  |  |  |  |  |  |  |  |  |  |  |  |  |  |  |  |  |  |  |  |  |  |  |  |  |
| 12. Isolate A538 | 8.5 | 4.5 | 8.1 | 5.2 | 6.0 | 4.9 | 2.2 | 7.2 | 8.7 | 5.7 | 0.1 |  |  |  |  |  |  |  |  |  |  |  |  |  |  |  |  |  |  |  |  |  |  |  |  |  |  |
| 13. Isolate A539 | 7.1 | 5.8 | 6.2 | 7.5 | 7.6 | 6.3 | 6.4 | 3.6 | 6.9 | 8.1 | 6.6 | 6.6 |  |  |  |  |  |  |  |  |  |  |  |  |  |  |  |  |  |  |  |  |  |  |  |  |  |
| 14. Isolate BIO-54 | 6.7 | 6.7 | 6.2 | 7.0 | 8.2 | 6.9 | 6.4 | 3.4 | 6.8 | 8.0 | 7.0 | 7.0 | 2.3 |  |  |  |  |  |  |  |  |  |  |  |  |  |  |  |  |  |  |  |  |  |  |  |  |
| 15. Isolate SU12 | 3.3 | 9.4 | 3.7 | 8.7 | 10.3 | 9.1 | 7.6 | 8.2 | 1.4 | 10.5 | 8.3 | 8.3 | 6.8 | 6.9 |  |  |  |  |  |  |  |  |  |  |  |  |  |  |  |  |  |  |  |  |  |  |  |
| 16. Isolate SU13.1 | 2.8 | 8.9 | 2.1 | 9.2 | 9.8 | 8.7 | 8.1 | 7.7 | 3.4 | 10.4 | 8.8 | 8.8 | 6.2 | 6.2 | 3.3 |  |  |  |  |  |  |  |  |  |  |  |  |  |  |  |  |  |  |  |  |  |  |
| 17. Isolate SU14.3 | 2.7 | 8.3 | 1.6 | 8.9 | 8.9 | 7.8 | 7.5 | 7.0 | 3.7 | 10.0 | 8.4 | 8.4 | 6.1 | 6.2 | 3.6 | 1.9 |  |  |  |  |  |  |  |  |  |  |  |  |  |  |  |  |  |  |  |  |  |
| 18. Isolate SU16.2 | 7.2 | 6.7 | 6.6 | 7.2 | 8.4 | 7.1 | 6.8 | 3.3 | 7.3 | 8.3 | 7.1 | 7.1 | 2.0 | 1.1 | 7.4 | 6.7 | 6.3 |  |  |  |  |  |  |  |  |  |  |  |  |  |  |  |  |  |  |  |  |
| 19. Isolate SU18.1 | 2.2 | 8.9 | 2.6 | 8.2 | 9.3 | 8.3 | 7.4 | 7.8 | 1.6 | 9.9 | 8.1 | 8.1 | 6.6 | 6.4 | 1.3 | 2.4 | 2.7 | 6.9 |  |  |  |  |  |  |  |  |  |  |  |  |  |  |  |  |  |  |  |
| 20. Isolate SU2 | 2.6 | 8.7 | 3.0 | 8.2 | 9.1 | 8.3 | 7.1 | 7.6 | 1.2 | 9.9 | 8.3 | 8.3 | 6.3 | 6.2 | 1.3 | 2.6 | 2.9 | 6.7 | 1.1 |  |  |  |  |  |  |  |  |  |  |  |  |  |  |  |  |  |  |
| 21. Isolate SU20.3 | 2.8 | 9.2 | 3.5 | 8.2 | 9.7 | 8.8 | 7.1 | 8.1 | 1.0 | 9.9 | 8.3 | 8.3 | 6.8 | 6.7 | 1.3 | 3.0 | 3.4 | 7.1 | 1.3 | 1.1 |  |  |  |  |  |  |  |  |  |  |  |  |  |  |  |  |  |
| 22. Isolate SU23.2 | 3.3 | 9.1 | 4.2 | 8.6 | 9.4 | 8.8 | 7.6 | 8.8 | 2.5 | 10.0 | 8.5 | 8.5 | 7.3 | 7.3 | 2.2 | 4.2 | 4.3 | 7.7 | 1.9 | 2.2 | 2.4 |  |  |  |  |  |  |  |  |  |  |  |  |  |  |  |  |
| 23. Isolate SU37.2 | 7.0 | 6.1 | 6.6 | 6.8 | 7.8 | 6.5 | 6.7 | 3.1 | 7.0 | 7.6 | 6.8 | 6.8 | 1.6 | 1.5 | 7.1 | 6.3 | 6.1 | 1.3 | 6.7 | 6.4 | 6.9 | 7.5 |  |  |  |  |  |  |  |  |  |  |  |  |  |  |  |
| 24. Isolate SU38.1 | 8.1 | 7.0 | 7.3 | 8.2 | 8.9 | 7.8 | 7.1 | 2.3 | 7.6 | 9.0 | 7.7 | 7.7 | 4.6 | 4.6 | 7.7 | 7.5 | 6.8 | 4.5 | 7.4 | 7.1 | 7.6 | 8.3 | 4.2 |  |  |  |  |  |  |  |  |  |  |  |  |  |  |
| 25. Isolate SU4.2 | 8.7 | 5.0 | 8.9 | 2.0 | 6.1 | 5.5 | 4.6 | 7.4 | 9.0 | 1.2 | 5.3 | 5.3 | 7.7 | 7.6 | 9.5 | 9.4 | 8.9 | 7.7 | 8.9 | 8.9 | 8.9 | 8.9 | 7.0 | 8.1 |  |  |  |  |  |  |  |  |  |  |  |  |  |
| 26. Isolate SU44.3 | 2.9 | 9.1 | 3.6 | 8.4 | 9.3 | 8.4 | 7.2 | 8.4 | 1.5 | 9.9 | 8.5 | 8.5 | 6.9 | 6.8 | 1.6 | 3.4 | 3.7 | 7.3 | 1.4 | 1.4 | 0.7 | 2.3 | 7.0 | 8.0 | 8.9 |  |  |  |  |  |  |  |  |  |  |  |  |
| 27. Isolate SU45.1 | 2.8 | 8.9 | 2.1 | 9.2 | 9.8 | 8.7 | 8.1 | 7.7 | 3.4 | 10.4 | 8.8 | 8.8 | 6.2 | 6.2 | 3.3 | 0.0 | 1.9 | 6.7 | 2.4 | 2.6 | 3.0 | 4.2 | 6.3 | 7.5 | 9.4 | 3.4 |  |  |  |  |  |  |  |  |  |  |  |
| 28. Isolate SU47.3 | 3.0 | 9.2 | 3.5 | 8.7 | 9.8 | 8.8 | 7.4 | 8.1 | 1.0 | 10.4 | 8.6 | 8.6 | 6.8 | 6.7 | 1.1 | 3.0 | 3.4 | 7.0 | 1.3 | 0.9 | 0.9 | 2.4 | 6.9 | 7.6 | 9.4 | 1.2 | 3.0 |  |  |  |  |  |  |  |  |  |  |
| 29. Isolate SU50.2 | 3.7 | 9.5 | 4.2 | 9.0 | 9.9 | 9.3 | 7.8 | 8.7 | 2.5 | 10.6 | 8.9 | 8.9 | 7.7 | 7.3 | 2.2 | 3.9 | 4.5 | 7.7 | 2.2 | 2.2 | 2.2 | 1.7 | 7.3 | 8.2 | 9.5 | 2.3 | 3.9 | 1.9 |  |  |  |  |  |  |  |  |  |
| 30. Isolate SU53.4 | 3.3 | 9.5 | 3.5 | 8.9 | 9.8 | 8.9 | 7.3 | 8.4 | 1.4 | 10.1 | 8.5 | 8.5 | 7.2 | 6.7 | 1.7 | 3.3 | 3.5 | 7.3 | 1.7 | 1.3 | 1.1 | 2.8 | 7.0 | 8.0 | 9.2 | 1.2 | 3.3 | 1.1 | 2.4 |  |  |  |  |  |  |  |  |
| 31. Isolate SU54.1 | 3.2 | 8.9 | 4.3 | 8.7 | 9.5 | 8.9 | 7.7 | 8.4 | 2.4 | 10.4 | 8.8 | 8.8 | 7.1 | 6.9 | 2.3 | 3.8 | 4.2 | 7.4 | 2.0 | 1.6 | 2.0 | 1.4 | 6.9 | 8.0 | 9.3 | 2.2 | 3.8 | 2.3 | 1.8 | 2.7 |  |  |  |  |  |  |  |
| 32. Isolate SU55.5 | 2.9 | 8.9 | 3.4 | 8.7 | 9.5 | 8.7 | 7.5 | 8.1 | 1.7 | 10.4 | 8.8 | 8.8 | 6.9 | 6.7 | 1.8 | 2.9 | 3.5 | 7.1 | 1.4 | 1.2 | 1.6 | 1.6 | 6.7 | 7.6 | 9.4 | 1.9 | 2.9 | 1.4 | 1.2 | 2.0 | 1.3 |  |  |  |  |  |  |
| 33. Isolate SU56.1 | 2.5 | 8.8 | 1.9 | 9.0 | 9.7 | 8.6 | 7.7 | 7.8 | 3.3 | 10.4 | 8.4 | 8.4 | 6.6 | 6.3 | 3.2 | 0.3 | 1.8 | 6.8 | 2.3 | 2.5 | 2.9 | 4.0 | 6.7 | 7.6 | 9.3 | 3.3 | 0.3 | 2.9 | 3.8 | 3.2 | 3.7 | 2.8 |  |  |  |  |  |
| 34. Isolate SU56.3 | 2.9 | 9.0 | 2.2 | 9.3 | 9.9 | 8.6 | 8.2 | 7.8 | 3.5 | 10.5 | 8.9 | 8.9 | 6.3 | 6.3 | 3.4 | 0.1 | 2.0 | 6.8 | 2.5 | 2.7 | 3.2 | 4.3 | 6.4 | 7.6 | 9.5 | 3.5 | 0.1 | 3.2 | 4.0 | 3.4 | 3.9 | 3.0 | 0.4 |  |  |  |  |
| 35. Isolate SU58.1 | 3.3 | 9.2 | 3.7 | 8.6 | 9.4 | 8.6 | 7.4 | 8.3 | 1.0 | 9.9 | 8.6 | 8.6 | 7.0 | 7.2 | 1.3 | 3.5 | 3.6 | 7.6 | 1.5 | 1.3 | 0.9 | 2.4 | 7.4 | 7.9 | 8.8 | 1.0 | 3.5 | 0.9 | 2.2 | 1.1 | 2.7 | 1.8 | 3.2 | 3.6 |  |  |  |
| 36. Isolate SU58.2 | 3.4 | 8.3 | 1.6 | 8.6 | 9.2 | 7.6 | 7.3 | 6.9 | 4.0 | 9.4 | 8.2 | 8.2 | 6.3 | 6.0 | 3.9 | 1.7 | 1.9 | 6.6 | 2.8 | 3.3 | 3.7 | 4.6 | 6.2 | 6.9 | 8.3 | 3.8 | 1.7 | 3.7 | 4.4 | 3.7 | 4.5 | 3.6 | 1.6 | 1.8 | 3.7 |  |  |
| 37. Isolate SU58.3 | 1.3 | 8.9 | 2.5 | 8.7 | 9.5 | 8.4 | 7.6 | 7.4 | 3.0 | 10.0 | 8.8 | 8.8 | 6.8 | 6.0 | 3.2 | 2.5 | 2.2 | 6.5 | 2.0 | 2.3 | 2.7 | 3.5 | 6.6 | 7.3 | 8.9 | 3.0 | 2.5 | 2.7 | 3.7 | 2.9 | 3.4 | 2.7 | 2.2 | 2.6 | 2.9 | 2.8 |  |
| 38. Isolate SU6.2 | 2.7 | 9.0 | 1.9 | 9.3 | 9.9 | 8.8 | 8.0 | 8.0 | 3.5 | 10.6 | 8.7 | 8.7 | 6.6 | 6.6 | 3.4 | 0.5 | 1.8 | 6.8 | 2.5 | 2.7 | 3.2 | 4.3 | 6.7 | 7.7 | 9.5 | 3.5 | 0.5 | 3.2 | 4.0 | 3.4 | 3.9 | 3.0 | 0.2 | 0.6 | 3.4 | 1.6 | 2.4 |
